# Studying human habit formation through motor sequence learning

**DOI:** 10.3758/s13415-025-01300-5

**Published:** 2025-05-06

**Authors:** Clarissa Carolin Grundmann, Viktoria Anna Arndt, Claudia Ebrahimi, Milena Philomena Maria Musial, Erik Lukas Bode, Florian Schlagenhauf, Tanja Endrass

**Affiliations:** 1 Addiction Research, Institute of Clinical Psychology and Psychotherapy, Faculty of Psychology, TUD Dresden University of Technology, 01062 Dresden, Germany; 2 Department of Psychiatry and Neurosciences | CCM, NeuroCure Clinical Research Center, Charité – Universitätsmedizin Berlin, Freie Universität Berlin and Humboldt-Universität zu Berlin, Berlin, Germany; 3 Faculty of Life Sciences, Department of Psychology, Humboldt-Universität zu Berlin, Berlin, Germany; 4 Einstein Center for Neurosciences Berlin, Charité – Universitätsmedizin Berlin, Freie Universität Berlin and Humboldt-Universität zu Berlin, Berlin, Germany; 5 Bernstein Center for Computational Neuroscience, Charité – Universitätsmedizin Berlin, Freie Universität Berlin and Humboldt-Universität zu Berlin, Berlin, Germany

**Keywords:** Habit, Motor learning, Implicit learning, Automaticity, Action slips

## Abstract

Habits are automatic behaviors triggered by specific cues and are thought to optimize daily activities by reducing cognitive effort and enabling efficient and fast performance. Yet, they can also lead to inflexibility, preventing individuals from adapting to environmental changes. Since it has been difficult to examine habit formation in humans with traditional outcome devaluation paradigms, we applied a motor sequence learning task (MSLT) to study this process. Thirty-one participants (16 female, 28.4 ± 5.3 years old) completed the MSLT on two consecutive days. They implicitly learned to execute a 12-item motor sequence using four fingers, each corresponding to one of four distinct visual stimulus locations. Test blocks introduced sequence deviations by intermittently omitting one item of the sequence. We measured whether participants were able to flexibly adapt their behavior or would incorrectly execute the omitted response – a so-called action slip. Action slips serve as an indicator of automatization or behavioral inflexibility. Findings indicate that prolonged training led to faster response times and lower error rates in learning compared to random blocks, suggesting successful sequence learning and the emergence of automatic behaviors. Action slips increased with extensive training, demonstrating the shift towards automatic and inflexible responding, indicative of habit formation. The results highlight the utility of the MSLT in studying habit formation in humans and emphasize the role of extensive training, motor skills, and automaticity. The task offers a promising framework for investigating the neural and cognitive mechanisms underlying habitual behavior, providing new insights into the balance between habitual and goal-directed control.

## Introduction

Habits play a crucial role in daily activities by minimizing the need for cognitive engagement and enabling efficient and automatic task performance. For example, tying shoelaces or shifting gears in a manual car requires little conscious attention due to habitual learning. While habits enhance efficiency and reduce errors, they can also lead to behavioral inflexibility, making adaptations to changes in the environment challenging. This difficulty is evident when switching from a manual to an automatic car for the first time, as ingrained stimulus-response (S-R) associations persist despite changing conditions.

Despite being useful in everyday life, habitual behavior is assumed to contribute to mental health problems, including substance use disorders (SUDs) or obsessive-compulsive disorder (Ersche et al., 2016; Lüscher et al., 2020; Voon et al., 2015; for a review, see Doñamayor et al., 2022). Behaviors that begin as being goal-directed can become rigid and automatic, reducing the ability to overcome learned stimulus-response (S-R) associations (Dolan & Dayan, 2013; Sjoerds et al., 2013). Animal and human research has demonstrated that neural circuits involved in the transition from goal-directed to habitual behavior are also involved in the development of substance use disorders (Balleine & O’Doherty, 2010; Giannone et al., 2024; Goldstein & Volkow, 2011; Hu et al., 2015; Kim et al., 2017).

Habits are defined as learned actions in response to specific situations (Robbins & Costa, 2017) and are characterized by a high degree of automaticity, cue sensitivity (S-R associations), and inflexibility (Orbell & Verplanken, 2010). They emerge through repeated reinforcement of cue-response associations. By contrast, goal-directed behavior involves knowledge about the relationship between a specific action and its outcome, allowing for flexible adaptation based on the contingency and desirability of the outcome (Dolan & Dayan, 2013; Doñamayor et al., 2022). While goal-directed actions are more adaptable, they also require greater cognitive resources.

Several tasks have been used to examine habit formation or the balance between goal-directed and habitual responding in humans, including the two-step task (Daw et al., 2011), contingency degradation tasks (e.g., Liljeholm et al., 2011; Shanks & Dickinson, 1991), and outcome devaluation tasks, for example, the fabulous fruit game or slips-of-action task (e.g., de Wit et al., 2007; Gillan et al., 2011). However, studies using these tasks have often struggled to reliably induce habits through extensive training (e.g., de Wit et al., 2018). These results suggest that such paradigms may not optimally capture habit formation. Therefore, some researchers have suggested alternative methods based on simpler response-outcome associations, such as repeated visuomotor responses (Hardwick et al., 2019; Tricomi et al., 2009) or motor sequence learning (Szegedi-Hallgató et al., 2017). In these tasks, participants train either sequences or specific mappings of visuomotor responses – like pressing buttons in response to certain visual stimuli – until these actions become both highly efficient and rigidly automatic (Doñamayor et al., 2022).

Motor sequence learning and the use of multi-finger tapping tasks have a well-established role in studying automatic behavior. In particular, investigating sensorimotor synchronization and how motor responses align with external rhythms has improved our knowledge of sequence acquisition and different mechanisms of error correction (Repp, 2005). In addition, Dezfouli and Balleine (2013) showed that sequential responding may also be observed in the two-step task. They demonstrated that once action sequences developed, combining first- and second-stage actions of the task, they were automatically executed as single response units and became insensitive to changes in the outcome value (Dezfouli et al., 2014). The authors concluded that habits are sequences of actions governed hierarchically by a goal-directed system. Although this approach suggests a link between sequential responding and habits, the sequences in the two-step task include only two responses. Our study focuses on longer, extensively trained motor sequences, and we probe them with the omission of single responses instead of devaluation of the entire sequence.

Motor learning represents a unique form of implicit learning and refers to the process by which repeated practice leads to increased efficiency and automaticity in executing motor responses (Seger, 1997). Both motor skill learning and habit formation rely on repeated practice and can develop over time into automatic actions that are performed without much conscious thought. Accordingly, motor skills as overly fixed behavioral routines can provide valuable insight into aspects of habit formation on a behavioral and neuronal level (Ashby et al., 2010; Doyon et al., 2018; Magon et al., 2020; Poldrack et al, 2005).

Our study aimed to develop a simple task for assessing habit formation by building on established principles of motor sequence learning (e.g., Desrochers & McKim, 2019). We used a motor sequence learning task (MSLT) that involves extensive training of a sequence of repeated visual stimuli combined with corresponding motor responses. With extended training, overcoming an automatic sequential behavior in favor of goal-directed behavior becomes increasingly challenging, especially under time pressure (Hardwick et al., 2019). Similar approaches, such as the Alternating Serial Reaction Time Task, have already been used to examine how individuals adapt to sequence changes (Szegedi-Hallgató et al., 2017). In this study, participants learned two distinct sequences, where anticipatory errors indicated interference effects between prior and newly learned sequences. In contrast, our study entirely focused on habitual behavior by assessing the degree of automatic responding through behavioral inflexibility.

We hypothesized that extensive training in the MSLT would lead to reduced response times and error rates in learning blocks compared to random blocks, indicating the automatization of behavior and successful sequence learning. We then tested behavioral flexibility by randomly omitting single stimuli within the learned sequence, requiring participants to adjust their responses accordingly. With prolonged training, we expected that overcoming these omissions would become increasingly difficult, leading to an increase in action slips. These action slips are erroneous responses directly triggered by the prior S-R or R-R associations (Du et al., 2022), and thus serve as indicators of inflexible, automatic, and habitual responding (de Wit et al., 2018; Hardwick et al., 2019).

## Methods

### Participants

Thirty-one participants were recruited from the Technical University of Dresden, Germany, and the surrounding community. Prior to participation, all participants underwent telephone screening to confirm their health status, ensuring they had no known history of neurological or psychiatric diagnoses. The age of participants ranged from 20 to 44 years (M = 28.4, SD = 5.3), with 16 of them being female (51.6%). Almost all participants had completed high school, except for one who finished tenth grade. Among the participants, 26 were right-handed, two were left-handed, and three reported being bi-manual. Participants gave written informed consent for both participation and data publication, and received 15 € or course credit in exchange for their participation. Ethics approval was granted by the Ethics Committee of the Technical University Dresden (EK 511122018). The sample size was determined to detect a within-subject effect size of d = 0.5 with a power of 80% at an alpha level of α=.05 in a one-sided t-test.

### Study design

**Fig. 1:**
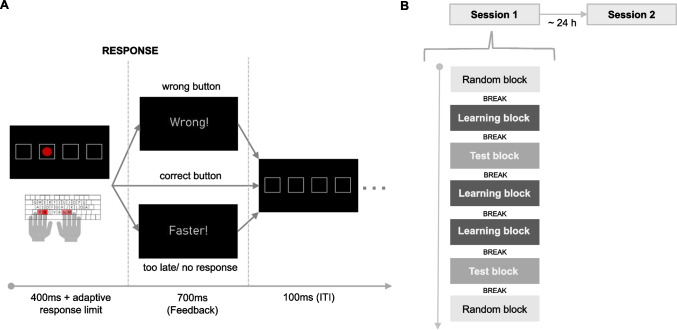
Study procedure and experimental design of the motor sequence learning task (MSLT). **A** Example trial of the MSLT. A red dot appeared for 400 ms at one out of four positions, designated by squares. Participants were asked to put their right and left index and middle fingers on four spatially corresponding buttons and to press the button corresponding to the location of the red dot within an adaptive response window. If a response was too slow or incorrect, a feedback screen appeared, displaying either “faster” (in German: “schneller!”) or “wrong” (in German: “falsch!”) for 700 ms. Following the button press or feedback screen, a 100-ms inter-trial interval (ITI) displayed four empty squares before the next trial began with the presentation of the red dot. **B** Study design. Participants performed the MSLT on two consecutive days, with the procedure being identical in both sessions. The MSLT featured three experimental conditions: random, learning and test blocks. Random blocks were performed at the beginning and the end of each session. Each block consisted of 99 trials featuring a pseudo-randomized sequence. Three learning blocks were presented, each consisting of 792 trials, including 66 repetitions of the 12-item sequence with 10-s breaks after every 264 trials. Test blocks were introduced after the first and third learning block. Each test block comprised 759 trials, which included 66 repetitions of the 12-item sequence. During every second sequence presentation, one position of the sequence was skipped, resulting in a total of 33 skip events. Additionally, a 10-s break was given every 253 trials. A self-paced break of at least 30 s was provided between task blocks

Participants took part in a two-session experiment conducted on two consecutive days, with both sessions occurring at approximately the same time of day. Each session started with general instructions followed by the motor sequence learning task (see Fig. 1). In the MSLT, participants manually responded to visual stimuli presented at four fixed positions on the screen in a 12-item sequence to facilitate implicit motor sequence learning (see Fig. 1A).

#### Motor Sequence Learning Task (MSLT)

Participants were first introduced to the task through an instructional block, which included detailed instruction across 12 trials, followed by a practice block consisting of 36 trials. The instructions emphasized the importance of responding to the stimuli both quickly and accurately by pressing the respective button. Participants were not informed about the sequential order of trials.

The task consisted of three experimental conditions: random, learning, and test blocks. In random blocks, 99 trials were presented pseudo-randomized. For learning blocks, 792 trials followed a fixed 12-item sequence that was presented consecutively. In test blocks, 759 trials followed the same 12-item sequence, but in every second sequence one item was skipped at differing positions and the subsequent item in the sequence was displayed directly. In order to hide the reoccurring nature of the sequence, the blocks started at a randomly selected position of the 12-item sequence. The duration of trials and time for responding was adjusted throughout the experiment. Each block began with a response limit set as twice the median of reaction times (RTs) during the practice block. This limit is then reduced by 30 ms after every three consecutive correct responses. If the response was incorrect, it increased to 1.5 times the median RT of the current block. Between condition blocks, a self-paced break of at least 30 s was shown, and within each condition block, two 10-s breaks were included to reduce fatigue during the task (see Fig. 1B).

The MSLT was presented using Presentation® software (Version 21.1, Neurobehavioral Systems, Inc., Berkeley, CA, USA; www.neurobs.com). The four buttons (Y, X, N, M) of a standard German keyboard from left to right were assigned to the middle and index fingers of the left hand and the index and middle fingers of the right hand, respectively (see Fig. 1A). Task duration differed depending on participants’ performance. The mean duration of the first and second session was 32 min and 26 min, respectively.

At the end of the experiment, participants were informed that the sequence of stimuli presented during the task followed a recurring pattern. They were then asked to generate this pattern or at least parts of it, if possible. Six participants were able to reproduce the entire 12-item sequence and thus became explicitly aware of the sequence during training. Five participants accurately reproduced ten or 11 items, three recalled between seven and nine items, ten reproduced five or six items, and another ten participants were only able to recall three or four items from the sequence.

### Statistical analyses

#### Motor-sequence learning

Data analyses were performed using Matlab 2020b and IBM SPSS Statistics 27. First, the analysis focused on evaluating whether participants had successfully learned the sequence, which was expected to result in reduced RTs and error rates in the learning condition compared to the random condition. To distinguish sequence-learning effects from general training effects, such as increased task familiarity, a three-way repeated-measures ANOVA (rmANOVA) was applied to the median RTs with the within-subject factors session (session 1 vs. session 2), block (first vs. last) and condition (random vs. learning). Note that, in the learning condition, the last block specifically referred to the third learning block (see Fig. 1B). Median RTs were calculated for each participant per block, condition, and session considering only correct responses and excluding errors related to timing or incorrect button presses. To test effects on error rates, we performed a three-way rmANOVA using the same within -subject factors as for RT analyses. Error rates were calculated as the rate of incorrect responses divided by the number of trials per block, condition, and session. Significant interaction effects were evaluated using Bonferroni correction for multiple comparisons. Corrections for violations of sphericity were done where appropriate using Greenhouse Geisser adjustments. Effect sizes were estimated using partial eta^2^ (${\eta }_{p}^{2}$).

#### Action slips as a measure of habitual behavior

Second, we aimed to investigate the development of automaticity and the formation of habit-like behaviors over the course of training. This was assessed through the occurrence of action slips, which were operationalized as errors made during sequence disturbances (i.e., skip-events) in the test condition, where the expected trial in a sequence was omitted, and the subsequent trial was shown instead. An action slip was defined as the participant’s response to the skipped trial, rather than the actual presented trial, indicating a reliance on the learned sequence. To analyze these action slips, we conducted a two-way rmANOVA with session (session 1 vs. session 2) and block (test block 1 vs. test block 2) as within-subject factors. The analysis used the total number of action slips per participant in each test block as dependent variable.

To quantify the increase in action slips across training on an individual basis, we additionally used the reliable change index (RCI) (Jacobson & Truax, 1991) to determine whether the change observed in an individual’s score across two time points is statistically significant, beyond what might be expected due to measurement error alone. The RCI is calculated using the following formula:

$$\text{RCI }= \frac{X2-X1}{\text{Sdiff}}$$

where:

X_1_ = represents the number of action slips in the first test block of session 1;

X_2_ = represents the number of action slips in the last test block of session 2;

S_diff_ = is the standard error of difference between these two scores and is calculated as:

$$\text{Sdiff }= \sqrt{2({\text{SE}}^{2})}$$

where SE (standard error of measurement) is derived from:

$$\text{SE}=SD \sqrt{\left(1- {r}_{xx}\right)}$$

where:

r_xx_ = is the reliability coefficient of the measure, which quantifies the consistency of the measurement across time.

SD = is the standard deviation of the number of action slips in the first test block of session 1.

An RCI equal to or greater than 1.96 corresponds to a reliable change in action slips from session 1 to session 2 which is not attributable to chance or measurement error alone.

## Results

### Motor sequence learning

#### Reaction times

**Fig. 2:**
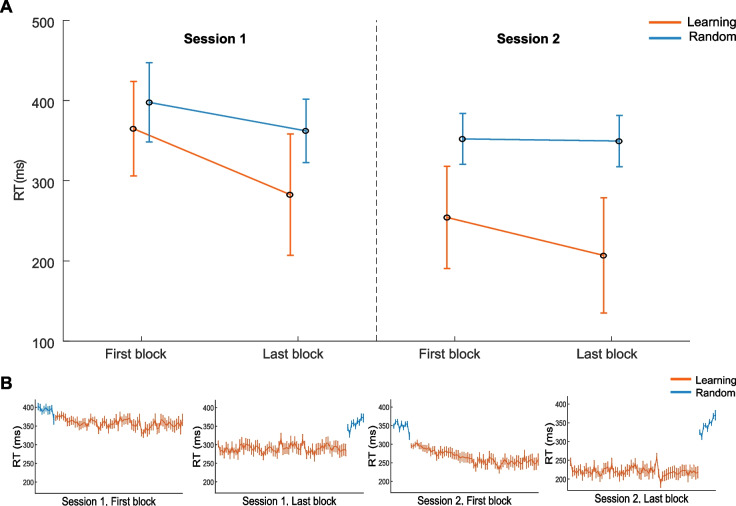
**A** Average median reaction times with standard deviations of the first and last block in session 1 and session 2 for both learning and random conditions. (**B**) Median reaction times (RTs) with standard errors binned every 12 trials, presented in the order blocks were performed during the experiment

RTs decreased from session 1 to session 2 across both conditions, indicating that participants responded faster in the second session. Within each session, RTs also decreased on average from the first to the last block, reflecting within-session learning. Notably, median RTs were consistently lower in the learning condition compared to the random condition (see Fig. 2A). These findings were supported by the three-way rmANOVA on RTs, which revealed significant main effects for session (*F*(1, 30) = 231.23, *p* < .001, ${\eta }_{p}^{2}$ = .89), block (*F*(1, 30) = 114.54, *p* < .001, ${\eta }_{p}^{2}$ =.79), and condition (*F*(1, 30) = 134.43, *p* < .001, ${\eta }_{p}^{2}$ = .82). For a detailed illustration of the change in RTs in both conditions within consecutive blocks, see Fig. 2B. Interaction effects indicated that the reduction in RTs was more pronounced in the learning condition compared to the random condition, both across and within sessions. From session 1 to session 2, the learning condition demonstrated a larger mean reduction in RTs (*M*_Diff_ (first-last) = 93.28, *SE* = 6.12) than the random condition (*M*_Diff_ (first-last) = 29.15, *SE* = 3.72). This was also evident within sessions, with the learning condition showing greater reductions (*M*_Diff_ (first-last) = 64.90, *SE* = 6.05) from the first to the last block than the random condition (*M*_Diff_ (first-last) = 19.16, *SE* = 3.56). This further suggests that participants learned the sequence, which allowed for greater improvement with practice compared to the random condition where no sequence was presented. These results were reflected by significant interaction effects between condition and session (*F*(1, 30) = 109.00, *p* < .001, ${\eta }_{p}^{2}$= .78) and between condition and block (*F*(1, 30) = 56.61, *p* < .001, ${\eta }_{p}^{2}$ = .65). In addition, the reduction in RTs from the first to the last block was more pronounced in session 1 (*M*_Diff_ (first-last) = 58.92, *SE* = 5.34) than in session 2 (*M*_Diff_ (first-last) = 25.14, *SE* = 3.86), suggesting that much of the learning occurred early in training. This was supported by a significant interaction between session and block (*F*(1, 30) = 45.48, *p* < .001, ${\eta }_{p}^{2 }$= .60). The three-way interaction between session, condition, and block was not significant (*F*(1, 30) = 0.05, *p* = .82).

#### Error rates

**Fig. 3:**
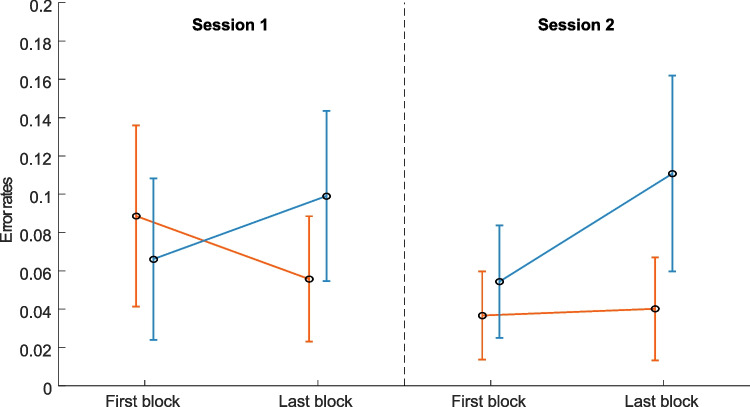
Average relative number of errors with standard deviations of the first and last block in session 1 and session 2 for both learning and random conditions

Overall, error rates decreased from session 1 (*M* = 0.077, *SE* = 0.006) to session 2 (*M* = 0.061, *SE* = 0.004) and were on average lower in the learning condition (*M* = 0.055, *SE* = 0.005) compared to the random condition (*M* = 0.083, *SE* = 0.006, see Fig. 3). This was supported by the three-way rmANOVA on error rates, which revealed significant main effects for session (*F*(1, 30) = 18.60, *p* < .001, ${\eta }_{p }^{2}$= .38), condition (*F*(1, 30) = 33.08, *p* < .001, ${\eta }_{p}^{2}$= .52), and block (*F*(1, 30) = 9.27; *p* = .005; ${\eta }_{p}^{2}$= .24). Interaction effects revealed that changes in error rates differed between conditions, both across and within sessions. Specifically, error rates in the learning condition showed a notable decrease from session 1 to session 2, indicating improvement over time, while error rates in the random condition did not change. A significant interaction between session and condition (*F*(1, 30) = 36.69; *p* <.001; ${\eta }_{p}^{2}$= .55) supported these findings, with post hoc analyses showing a significant reduction in error rates from session 1 to session 2 in the learning condition (*t*(30) = 7.83, *p* < .001), but not in the random condition (*t*(30) = 0.00, *p* = 1.00). Within sessions, error rates also decreased from the first to the last block in the learning condition, but increased in the random condition. This was reflected by a significant condition and block interaction (*F*(1, 30) = 109.53, *p* < .001, ${\eta }_{p}^{2}$= .79), with post hoc analyses showing a significant decrease in error rates from the first to the last block in the learning condition (*t*(30) = 3.65 *p* < .001), contrasting with an increase in the random condition (*t*(30) = −6.42, *p* < .001). The significant interaction between session and block (*F*(1, 30) = 13.72, *p* < .001, ${\eta }_{p}^{2}$= .31) further demonstrated that the overall change in error rates from the first to the last block differed across sessions. While error rates remained stable from the first block to the last block in session 1 (*t*(30) = −0.003, *p* = .998), they increased in session 2 (*t*(30) = −5.73, *p* < .001). This effect was primarily driven by higher error rates in the random condition, suggesting that the absence of a sequence made it more challenging for participants to maintain consistent performance across blocks. The three-way interaction between session, condition, and block was not significant (*F*(1, 30) = 1.68, *p* = .20).

### Action slips as a measure of habitual behavior

**Fig. 4:**
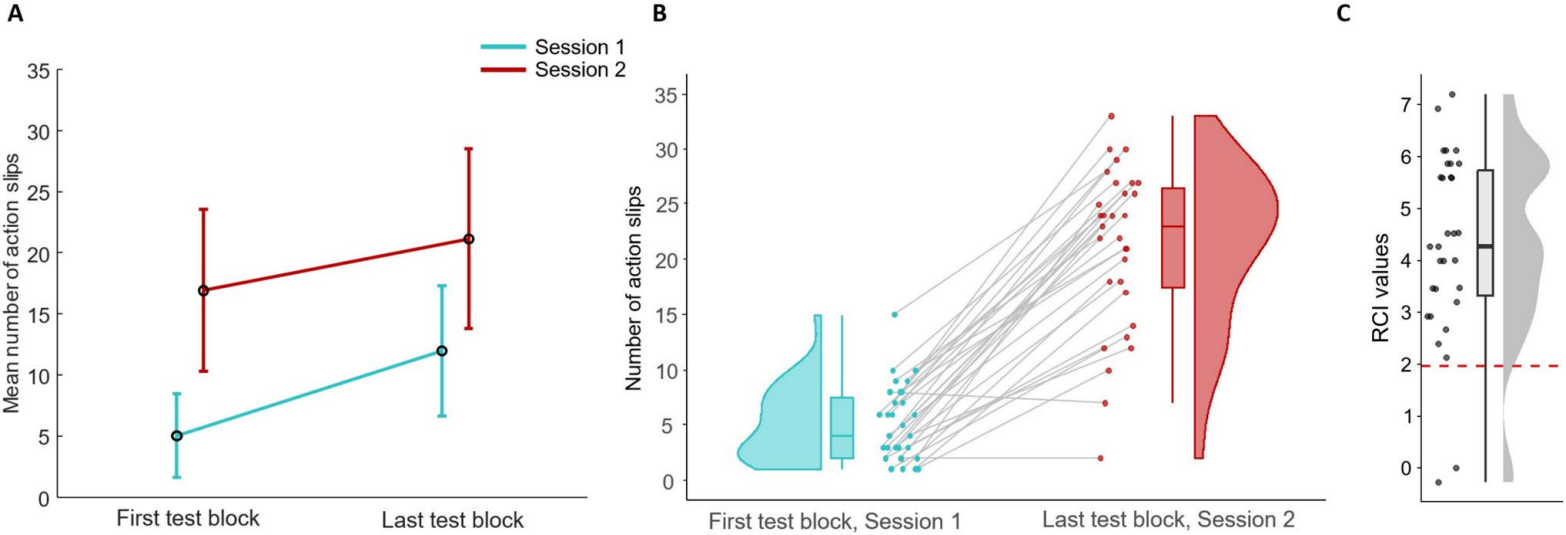
**A** Mean number of action slips (max. 33) separately for both sessions and test blocks. Error bars represent standard deviations. **B** Distributions, box-plots and individual scores of action slip data for session 1 (first test block) vs. session 2 (last test block). Grey lines connect participants' data from session 1 (first block) to session 2 (last block). **C** Individual reliable change index (RCI) of action slips from session 1 (first block) to session 2 (last block). Values above the cut-off line at 1.96 are considered significant

The analysis of action slips revealed an increase in the number of action slips between session 1 and session 2, as well as an increase between the first and last test block within each session (see Fig. 4). The difference in the number of action slips from the first to the last block varied between sessions, with the increase being more pronounced in session 1 (*M*_Diff_ (last-first) = 6.94, *SE* = 0.79) than in session 2 (*M*_Diff_ (last-first) = 4.23, *SE* = 0.78). This was supported by a two-way rmANOVA, which yielded a significant main effect of session (*F*(1, 30) = 139.31, *p* < .001, ${\eta }_{p }^{2}$= .82) and block (*F*(1, 30) = 115.37, *p* < .001, ${\eta }_{p }^{2}$= .79), as well as a significant interaction between session and block (*F*(1, 30) = 5.33, *p* = .028; ${\eta }_{p }^{2}$= .15). Together, these findings indicate that participants made more errors and that the behavior became more habitual as training progressed, leading to more automatic responding to sequence disturbances.

The RCI calculation for the increase in action slips from the first test block of session 1 to the last test block of session 2 showed that 93.5% of participants had a significant increase in action slips with an RCI > 1.96 (see Fig. 4C). This finding, illustrated in more detail in Fig. 4B, underscores the robustness of the effect induced with our task across the participant pool, suggesting that the observed increase in action slips is a general trend among the majority of participants.

While the number of action slips increased, median RTs decreased across the four test blocks, starting with the highest median RT in the first test block of session 1 (*Mdn* = 343 ms, *SD* = 49.12), followed by the last test block in session 1 (*Mdn* = 301 ms, *SD* = 46.52). Similarly, RTs continued to decrease in session 2, first test block (*Mdn* = 269 ms, *SD* = 44.73), and session 2, last block (*Mdn* = 251 ms, *SD* = 46.34). We calculated Pearson correlations to examine the association between the number of action slips made by participants and their average RT for each test block. The results revealed strong negative correlations between the median RTs and action slips across all test blocks, providing evidence for a speed-accuracy trade-off where faster RTs are strongly associated with an increased likelihood of action slips (session 1, test block 1: *r* = −.58, *p* < .001; session 1, test block 2: *r* = −.79, *p* < .001; session 2, test block 1: *r* = −.84, *p* < .001; session 2, test block 2: *r* = −.80, *p* < .001). This supports the hypothesis that habitual responding is more likely under time pressure (see Hardwick et al., 2019).

## Discussion

This study examined habit formation and behavioral flexibility through a motor sequence learning paradigm. Participants learned a fixed 12-item motor sequence over two consecutive days, allowing for consolidation between sessions. Each session consisted of learning blocks and test blocks with omissions of single items of the sequence to challenge automatic behavior. Our findings indicate that extensive training led to increased response speed, accuracy, and resistance to change, reflected in automatized behavior. Performance improvements in RTs and error reduction were observed within and across sessions, suggesting training effects that are not merely due to task familiarity. In test blocks, sequence disturbances through skip-events resulted in an increased number of action slips with prolonged training, underscoring the increased automaticity and reduced flexibility associated with habitual responses.

Our findings align with previous research on the learning of motor sequences and repeated visuomotor responses (Frölich et al., 2023; Hardwick et al., 2019; McKim et al., 2016). These studies showed that extensive training enhances automatic responding, with participants demonstrating faster RTs and higher accuracy over time, indicative of habit formation, mostly at the expense of flexibility. Hardwick and colleagues (2019) found that habitual responding increased under time pressure. This highlights the interaction between extensive practice and the time available to respond, influencing the balance between habitual and goal-directed actions. McKim et al. (2016) explored habitual tendencies with a relearning task (after successful initial learning) and observed greater habitual errors in individuals with a history of SUDs compared to controls. Frölich et al. (2023) further demonstrated that learned sequences interfere with goal-directed choices under conflicting conditions in dual-target trials. However, instead of altering the S-R mapping or using dual-target trials to measure errors as an indicator of habit formation, we employed skip-events in a practiced sequence to provoke action slips. Thus, our results extend these findings by highlighting the role of action slips as a further indicator of habitual responding.

Nevertheless, studying habit formation in humans in a laboratory setting has yielded mixed findings. While some studies successfully induced habitual behavior (Hardwick et al., 2019; Tricomi et al., 2009), others like de Wit et al. (2018) encountered difficulties using different outcome devaluation paradigms. These paradigms assess whether behavior remains goal-directed (i.e. sensitive to devaluation) or becomes habitual after extensive training (de Wit et al., 2018). However, it has been suggested that inconsistent findings may arise from task complexity obscuring habit formation. Recent studies advocate for simpler methodologies, such as fixed action sequences to better capture habitual responding (Dezfouli et al., 2014; Frölich et al., 2023). Our study supports these approaches by demonstrating robust habitual behavior through a sequence learning task. The Reliable Change Index demonstrated that 93.5% of participants showed significant increases in action slips, underscoring response inflexibility.

Unlike traditional habit paradigms, our approach aligns with research suggesting that habit formation is also possible without rewards during the acquisition phase (Hogarth et al., 2019; Miller et al., 2019). Specifically, the study by Luque et al. (2020) focused on measuring habit formation through identifying RT switch costs after operant training. They demonstrated that RT switch costs increased with overtraining and suggested this as a more nuanced marker of habitual responding than traditional measures, which typically assess response selection for a devalued outcome. Action slips in our study reflect a conflict between habitual and goal-directed systems, where participants struggled to adapt learned responses when sequences were disrupted. Increased training made adaptation more difficult, leading to habitual responding.

Based on the results by Hardwick et al. (2019), our study incorporated time pressure into the design of the MSLT. The action slips in our task served as indicators of the rigidity of sequence execution after extensive training, which became particularly evident when behavioral responses were limited in time. Du et al. (2022) explored the link between habits and motor skills, proposing that action slips may represent habitual tendencies associated with skill acquisition due to the necessity of rapid stimulus-driven action selection. The efficiency of this rapid selection process facilitates a speed-accuracy trade-off, at the expense of flexibility. Our findings suggest that after extended training, participants’ behavior became increasingly automatic but also more inflexible, particularly in conditions requiring adaptive stimulus-response selection. We applied a 2-day training, specifically designed to include the potential influence of overnight sleep on habit formation (Debas et al., 2010). Training sessions were roughly 24 h apart to combine immediate training effects with the impact of memory consolidation, but we cannot delineate the influence of these aspects individually, as we did not systematically vary them. Future studies should further investigate the interaction of consolidation and practice, as suggested by Robertson et al. (2004). They emphasize that off-line improvement, which occurs between practice sessions, and memory stabilization are key aspects of procedural consolidation.

Habits and skilled actions exhibit greater automaticity (Lally et al., 2010), often resulting in behavioral inflexibility due to overtraining, but they also promote efficient use of cognitive and attentional resources, allowing effortlessness and rapid responding (Du et al., 2022; O’Hare et al., 2018). Drawing from skill acquisition literature, we see a significant overlap between habits and skills, especially in the realm of motor skills. Motor skills require significant practice and are designed for precise and fast execution, aligning with the essence of habitual behavior. Although the purpose of motor skill development is to maintain adaptability within specific contexts, thereby diverging from habitual tendencies, motor skills combine both cognitive (adaptive) and automatic (rigid) processes. This interplay allows us to leverage the automatic properties of motor skills to investigate habit formation (Pacherie & Mylopoulos, 2021). Both habits and motor skills are attained through frequent practice, gradually reinforcing S-R associations until they become highly automatic but inflexible, underscoring the close relationship between habitual behaviors and automaticity in skilled performance (Lally et al., 2010).

Integrating insights from habit research and skill research is useful for delineating the limitations and distinctions of both constructs. Despite sharing features like automaticity and extensive training, habits and skills are fundamentally different in certain aspects, crucial for understanding the nuances of habit formation through the lens of motor skills. One key difference lies in the flexibility of skilled performance versus the rigidity of habitual behaviors. For instance, a highly skilled musician can adapt their playing to various musical styles or respond creatively to unexpected events during a performance, showcasing the flexibility inherent in skilled actions. Conversely, habitual behavior, such as shifting gears while driving, is performed automatically without thinking about alternative approaches and without representing the desired outcome. Research often operationalizes habits as a basic S-R association, a simplification that has been criticized (e.g., see Lally et al, 2010; Vandaele & Ahmed, 2021). This raises concerns about the applicability of laboratory findings to complex real-life behaviors, which are rarely driven by isolated stimuli and often involve multifaceted situational factors (Du et al., 2022). Bridging the gap between controlled laboratory experimental paradigms and complex real-life habits and skills remains a significant challenge for future research. Our findings highlight the utility of motor sequence learning for studying habit formation with implications for understanding automatic or habitual behaviors in contexts such as addiction. Future research should also investigate the neural correlates of motor automatization and action slips to advance our understanding of the transition from goal-directed to habitual behavior and how these complex processes relate to pathological behaviors.

## Data Availability

The data and materials for this experiment are available at https://osf.io/trxz7/.

## Code availability

The code for this experiment is available at https://osf.io/trxz7/.

## Declarations

### Open Practices Statement

The data and materials for this experiment are available at https://osf.io/trxz7/. This experiment was not preregistered.
